# The narcissism of minor resemblances: searching for allies at times of threat

**DOI:** 10.3389/fpsyg.2024.1332025

**Published:** 2024-06-26

**Authors:** Boris Drožđek

**Affiliations:** De Hemisfeer, Den Bosch, Netherlands

**Keywords:** enemies, allies, narcissism, minor differences, ethnic conflict, threat, political psychology, psychological trauma

## Abstract

Humans must identify others as enemies or allies to develop, protect, maintain, and refine their sense of self. This is a part of their normal psychological development. These phenomena operate on individual and large group levels and are pronounced under threat. In peril, they help create psychological boundaries between conflicting parties and bonds between allies. These boundaries and bonds are invested with strong emotions. The narcissism of minor differences concept is involved in identifying and delineating enemies at times of perceived danger. This article introduces the concept of the narcissism of minor resemblances. This concept is discussed from the psychodynamic perspective and illustrated with examples of socio-political developments from modern history. The narcissism of minor resemblances concept may help us understand the underlying dynamics of bonding with allies and identifying with others when undergoing threat and hardship. This concept connects the public arena of political action with individual psychological development. Awareness of this phenomenon can help mitigate the negative aspects of rigid enemy-ally distinctions and promote cooperation and peace. It may also help individuals impacted by psychological trauma to make meaning of psychological and societal processes experienced and contribute to their healing.

## Introduction

### On narcissism

The concept of narcissism is one of the most complex and confusing ones in psychoanalysis (Pulver, 1970). Throughout time, it has been applied to a range of issues, including a self-oriented motivational state, a normal phase of psychological development, a configuration of personality traits, and a personality disorder. A popular image of a narcissist is one of a vain and boastful, while a secretly insecure and ashamed person (Bosson et al., 2008) who perceives his/her own needs and goals as more significant than those of others.

Krizan and Herlache (2017) have recently developed the narcissism spectrum model as an integrative theoretical account. In this model, narcissistic traits were framed as diverse combinations of distinct and conflicting approach-oriented (i.e., bold and grandiose) and avoidance-oriented (i.e., reactive and vulnerable) qualities of entitled self-importance. Grandiosity and vulnerability exist side by side in the narcissistic personality and are expressed to varying degrees. Deep-seated feelings of inferiority are masked with surface-level feelings of superiority that keep the narcissistic person unaware of self-loathing and require continual reinforcement (Bosson et al., 2008). Other scholars (Weidmann et al., 2023) have proposed a trifurcated perspective on narcissism consisting of agentic (narcissistic admiration), antagonistic (narcissistic rivalry), and neurotic (vulnerable) aspects.

### Narcissism of minor differences

Identifying threats and forming alliances with others is essential for the survival of humankind. People need to recognize potential dangers to protect themselves and their communities. They also need to identify with others to create a sense of belonging and security, boost self-esteem, foster social cohesion, and provide a framework for understanding the world. The categorization of others into friends or foes is not steady. It can be fluid, context-dependent, and may sometimes seem irrational. Moreover, it can also be used as a vehicle for manipulation in political action, and can facilitate sowing fear in society and discharging aggression onto the “other.”

The term narcissism of minor differences was coined by Freud in 1917 (Freud, 1953a) and elaborated upon in 1921 (Freud, 1953b). The idea behind this concept is that individuals and groups with shared characteristics, values, and backgrounds may emphasize slight distinctions between them to assert their uniqueness and superiority. Freud also suggested that the antipathy of the narcissism of minor differences does not arise as a consequence of difference, but in the creation of difference, in the exaggeration of its presence. Minor differences gain importance at times of perceived threat. They may fuel hatred between individuals and large groups in a conflict that might seem disproportionate given the overall similarities.

A benign form of this phenomenon occurs in interpersonal relationships. For example, two colleagues have a heated discussion about an unimportant professional issue. Although their initial disagreement was not very important, they continue displaying arguments to support their opposing perspectives, thereby losing awareness of the discussion’s triviality (Werman, 1988). Another example of a benign form of the narcissism of minor differences is jokes wherein members of the “other” group are stereotyped and made fun of. Think of jokes made by English about Scots, or Aryan about Semites.

Unlike his other theories, Freud did not develop this one further, leaving many questions unanswered. For example, he has not elaborated in-depth on whether minor differences can cause animosity and conflicts between people or that they are just used to rationalize hostility (Kolsto, 2007).

The narcissism of minor differences phenomenon has been discussed later by psychoanalysts and social scientists trying to understand societal dynamics behind large-scale violent conflicts, like wars and genocide. Volkan (1985) elaborated upon Freud’s theory and concluded that having enemies and allies is a basic human evolutionary need connected with the developmental processes in childhood. By age 3, a child should complete uniting “good” and “bad” object representations and opposing drive derivates attached to them and achieve an integrated self-concept, integrated object representations, and tamed expression of drives. In cases where this does not occur, unintegrated “bad” object representations threaten a sense of self and need to be put “out there,” projected on the “other,” to enhance a sense of self. The exact process may unfold in adult individuals perceiving that their sense of self is threatened.

Moreover, when individual identity is in peril, it seeks protection under the umbrella of the collective identity of a large group, ethnicity, or nationality. Individual identity regresses and becomes subordinated to the collective one. When a large group feels threatened, it may also project unwanted parts of its identity onto an enemy and contaminate the enemy group with its negative mirror image. This process can become malignant and fuel large-scale conflicts and violence, dehumanization of the enemy, and its extermination. When the enemy is a neighbor resembling the threatened group in many ways or a member of the same large group, minor differences obtain a major psychological significance. They are magnified to strengthen the psychological gap between the opposing groups. They help increase cohesion within a group by delineating it from outsiders and direct aggression toward them (Volkan, 1986).

Figlio (2018) suggested another take on the narcissism of minor differences and focused on the dread of sameness rather than on antipathy toward the differences. It seems logical that humans stick together with others who are like them, and that they feel antipathy towards those who are different. However, Figlio (2018) argued that antipathy is more rooted in sameness than in difference, and suggested that a drive to be the same is an essential feature of narcissism. This drive is ambivalent. The urge to merge goes along with the hatred of it, and the conflicting illusions of omnipotence and helplessness exist here side by side. Therefore, violence is suggested to be inherent in sameness. The less difference between members of the same group, the more pronounced the narcissism of minor differences will be, and the higher the likelihood of a conflict.

Based on Volkan’s (1985, 1986) and Figlio’s (2018) perspectives, it can be argued that when a large group’s identity is eroded, unease and discomfort arise. A rift between the ego and the ego ideal occurs, and the group ego ideal has to be defended. One way to do this is to create an external enemy as a repository for projecting and discharging the large group’s aggressive fantasies. The narcissism of minor differences helps create the scapegoat. It does not arise as a consequence of difference but is created to make a difference. A wounded large group narcissism seeks soothing and regresses to the paranoid-schizoid position (Rhode, 1994). Omnipotent illusions of “all good” and “all bad” part-objects are split. The “all bad” part is projected onto the enemy and experienced as threatening to the group identity, while the “all good” part is identified in the ally and glorified. At the core of the narcissism of minor differences lies an unease in an individual or a large group, The challenge is not to manage differences, but the endogenous unease in a society (Figlio, 2018).Some examples from humankind’s history show how the narcissism of minor differences is involved in large group psychology at times of perceived danger and how it mediates malignant social developments.

In 1937, the Parsley massacre occurred in the Dominican Republic (Turits, 2002). On the orders of Dominican dictator Rafael Trujillo, the Dominican Army troops massacred many of the Haitian population living in the Dominican frontier regions in response to reports of Haitians stealing cattle and crops from Dominican borderland residents. The massacre claimed the lives of an estimated 14,000–40,000 victims, all men, women, and children, and lasted between 5 and 8 days. The two groups inhabit the same island and share a similar ancestry, but are divided by their different colonial pasts and they speak different languages (Spanish and French). The Dominican troops interrogated thousands of civilians in the border region, demanding that each person say the word “parsley” (*perejil* in Spanish). If one could not pronounce it with a rolling, Spanish r, he/she was deemed Haitian and killed. A minor difference (the language of the colonial power) between the two peoples on the same island was inflated into a significant marker of difference between them and was associated with massive bloodshed.

Between 1975 and 1979, more than 1 million Cambodians died during Pol Pot’s rule due to forced labor, hunger, disease, torture, and executions. Pol Pot wanted to establish a medieval agricultural state. All cities were emptied, and the population had to work in fields. Higher-educated Cambodians, such as teachers, were immediately killed; sometimes, wearing glasses was enough. Wearing glasses symbolized higher education and opposing political views. This minor difference was invested with strong emotions of aggression and used as the marker in the “auto-genocide” in this country (Charot, 2002).

In 1994, genocide took place in Rwanda. An estimated 500,000 to 1 million Tutsis (about 70% of the total population) and moderate Hutus were murdered by the Hutu militia over 100 days. Hutus and Tutsis speak the same language and share the same cultural background and belief system. The only differences between them are their body heights and traditional economies. While one group is engaged in agriculture, the other is breeding livestock. The colonial rulers previously provided this division of roles and considered Tutsis superior and privileged over Hutus (Mann, 2005). These minor differences between the two peoples were involved in one of the bloodiest genocides in modern history.

## The narcissism of minor resemblances

The narcissism of minor differences serves the delineation of the enemy when an individual or a large group feels frightened, angry, and has undergone narcissistic humiliation (Werman, 1988). However, the opposite may also be the case. Searching for allies while feeling vulnerable may also reduce feelings of anxiety and discomfort by expanding the boundaries of individual and collective identities. The narcissism of minor resemblances can be introduced as a new concept helping to explain how searching for an ally operates on individual and large group levels.

The narcissism of minor resemblances can operate benignly like that of minor differences. However, it can also facilitate malignant aggression under certain circumstances. Both processes will be described in the following examples.

A white man walks the streets of Tokyo as a tourist. He feels uprooted and far from home, somehow vulnerable and anxious. Everything around him is different. He does not speak Japanese, cannot read Kanji characters, and there are just a few directions in English on the streets. To find a restaurant or store he is looking for takes a lot of effort. People he asks for directions do not speak English. He has no acquaintances in town and walks alone. Once he encounters another white man in the street, chances are the two will greet each other, something people in a megalopolis usually do not do. But now, they may do so because they are both of the same race and feel different and alienated in Tokyo. If the other man is in a similar frame of mind and the opportunity presents itself, it is also possible that the two will start talking to each other. Maybe they will even have a drink together, get closer to each other, and “fraternize.” The narcissism of minor resemblances may be at the root of this “fraternization.” However, in conversation, they may find out that apart from their skin color and the *lingua franca* (English, which is not their native language), there are few other similarities between them. These two men might never communicate with each other in different circumstances. Still, confronted with feelings of estrangement and discomfort, they may seek each other’s company to feel safer and more relaxed.

In 2014, the annexation of Crimea took place, and the Russian army crossed the border into Ukraine to support separatists in the Donetsk and Luhansk regions of eastern Ukraine. Trench warfare ensued and has stood ever since without much line difference. NATO and the Dutch state, being a part of it, have accused Russia of invading Ukraine (North Atlantic Treaty Organization, 2014). No military action followed, and economic sanctions were imposed upon Russia. Later in the same year, Malaysia Airlines Flight 17 (MH17) was shot down by the Russia-controlled forces in eastern Ukraine. The crew and all the 283 passengers were killed. The majority of them were Dutch citizens. Following this incident, perceived in the Netherlands as a collective trauma, a series of national commemoration ceremonies and the building of the national MH17 monument occurred. The Dutch government started a thorough legal investigation of the accident and, in 2022, sentenced the accused *in absentia* to life imprisonment (Netherlands Public Prosecution Office, 2023).

In 2016, Dutch citizens could vote in a referendum on Ukraine’s accession to the Association Agreement with the 28 member states of the European Union (EU). Only 32.2% of Dutch citizens participated in this referendum, voting 61% against the accession (Rijksoverheid, 2016).

However, when the large-scale war between Russia and Ukraine broke out in 2022, the public perception of the Ukraine conflict changed dramatically in the Netherlands. People suddenly became in solidarity with Ukraine; Ukrainian flags were spontaneously hung up on many buildings in Dutch villages and cities, and people considered Ukrainian soldiers fighting not only for the liberation of their own country but also for democracy and freedom like those in the EU. The dominant societal narrative of the war in Ukraine was that of its soldiers defending the EU from Russian aggression, as Ukraine has been considered the attacked outer boundary of the EU values (Politi et al., 2023). The aspiration of Ukraine to become a democratic society and the admiration for its bravery in confronting Russia may have been the minor resemblances between the Dutch and the Ukrainian peoples. It has helped mobilize public support for large-scale Dutch and EU humanitarian and military aid to Ukraine, which has continued ever since. The Netherlands warmly welcomed refugees from Ukraine who are subject to different, less restrictive legal rules than asylum seekers and refugees from other war zones (Dahinden, 2022). As of August 2023, the total number of Ukrainian and Russian troops killed or wounded in the war was nearing 500,000 (Cooper et al., 2023). The war has resulted in Europe’s fastest-growing refugee crisis since World War II, global food shortages, and adverse effects on the world economy (Behnassi and El Haiba, 2022; UN High Commissioner for Refugees, 2022).

From a psychodynamic point of view, the narcissism of minor resemblances seems to occur unconsciously through identification in which an individual or a group assimilates positive images and functions of another within the self. Thereby, large groups also project ego-syntonic parts of the self onto allies and ego-dystonic ones onto enemies.

## Discussion

It has been argued that the narcissism of minor differences has been insufficiently elaborated in psychoanalysis because of its triviality (Werman, 1988). The same may apply to the narcissism of minor resemblances, previously not even coined as a term. However, both concepts can be valuable for the understanding of individual and large-group psychology at times of perceived uncertainty and threat.

In all the examples presented, the narcissisms of minor differences and resemblances can be perceived as vehicles, “reliable identifiers” of the enemy and the ally groups. The minor differences can be identified along ethnic lines, as illustrated by the examples from the Dominican Republic and Rwanda, along social lines, as found in the Cambodia example, and along economic, political, racial, cultural, and other affiliations. The narcissism of minor resemblances may also operate along racial lines, like in the Tokyo example, along ideological lines, like in the Ukraine war example, and others.

Kolsto (2007) has argued that hostility between opposing groups emerges due to a fight for power/status, economic interests, commodities, or territory. These causes are also the roots of conflicts presented in this article. Large-scale hostilities start upon a political decision. However, they can be sparked by a narcissistic injury perceived by the political elite and resonating with the emotions of a large group. The minor differences and resemblances are involved in the process later on, at the beginning of a conflict. Figlio (2018) pointed out that discomfort within a large group’s self and identity and a large group’s narcissistic injury may lie at the core of the creation of the narcissism of minor differences. Narcissistic ego ideal can be repaired through a painful process of self-examination. However, projecting the unwanted parts of the ego onto an enemy and fighting it is, psychologically, a much easier option. I argue that large-scale hostilities unfold when the “objective” aims of a conflict are supported by a “fertile” psychological constellation within a large group. Psychological mechanisms help shape the emotional involvement of a large group in a conflict, a conflict’s narrative, the rationalization of hostilities (Werman, 1988), and the motivation of large group members to participate in it.

Reflecting further on the Ukraine example, some critical processes have possibly facilitated the conflict. These were Russia’s loss of importance and power in international relations, an erosion of the United States importance in world politics and economy, the relative loss of national identities of the European states due to the EU unification, and a range of global sociopolitical developments, including mass migrations, post-COVID economic crisis, and climate change. All these processes have in common that they have caused narcissistic injuries to the respective large groups involved in the conflict, and have enhanced otherwise feelings of uncertainty and unease. A fertile soil for conflict in a psychological sense has emerged.

Regarding the involvement of the narcissism of minor resemblances in the conflict, the similarities between the EU, the Dutch, and the Ukrainian societies have objectively not increased since the Dutch referendum in 2016. These are nations of the same race but with different histories and cultures. However, they both seem to share the fear of Russian aggression rooted in world history. In the past, the dividing line between the West and the former Soviet Union was the antagonism between capitalism and communism. Upon the collapse of the Soviet Union, a new dividing line was established; the one between the Western democratic values stressing respect for human rights and the Russian totalitarian and autocratic society.

When a political decision to start the war was taken, the EU and the Dutch large groups seemed to have identified with the aspirations of the Ukrainians to become a society based on Western societal norms and values and to fight against Russia. Ukrainians were psychologically (re)categorized from the outgroup to the ingroup members of the EU. The psychological boundaries of the EU identity have expanded to Ukraine and identity fusion occurred (Politi et al., 2023). The EU and the Dutch large groups have projected their cultures’ “good” object representations onto Ukraine. At the same time, Russia became the repository of the “all bad” projections. European unity was revitalized in the face of a common enemy (Politi et al., 2023), and the involvement in the conflict unfolded according to the principle “the enemy of our enemy is our friend.” Displaying Ukrainian flags in many cities and villages across the EU can be perceived as a marker of collective regression, the appearance of ritualistic use of symbols to create a link between individuals within a large group entity (Volkan, 1985). The identifications and projections involved may mend, at least temporarily, the wounded narcissism of the EU and the Dutch societies. However, a collective regression to the paranoid-schizoid position incurs a risk of escalating large-scale violence. Thereby, internal identity problems within the EU and the Dutch societies will remain unaddressed. The narcissisms of minor differences and resemblances seem to serve as a defense mechanism against self-examination of narcissistic injuries.

Reflecting on the narcissism spectrum model (Krizan and Herlache, 2017), it can be argued that the narcissism of minor differences serves as an antidote for the grandiose aspect of narcissism, while the narcissism of minor resemblances soothes the vulnerable aspect. Therefore, both phenomena seem to be involved at the same time in the process of reparation of narcissistic injury to a varying degree when the large group self-regresses to the paranoid-schizoid position.

Moreover, it can be argued that social media also plays an important facilitating role in conflicts nowadays. Modern Western society is considered anthropocentric, highly individualized, and immersed in the influential media world. This world seems to be characterized by superficiality and emphasizes the emotional perception of events around us at the expense of a perspective based on knowledge, insight, and reason. The virtual space of the Internet and social media inundates the public with information about political events around the world with unprecedented immediacy. The availability and quantity (but not necessarily quality) of information can make one feel that the world is becoming increasingly unsafe and violent. It enforces and spins off feelings of anxiety, insecurity, and impending doom in individuals and societies. A recent study of psychological well-being in Europe after the outbreak of war in Ukraine (Scharbert et al., 2024) suggested a reciprocal relationship between daily well-being and salience. Individuals worldwide posted more war-related tweets on days when they were particularly distressed by it, and they became more distressed by being exposed to war-related content on social media.

Awareness of psychological processes within a large group contributes to the understanding of social and political developments. It may shed light on the seemingly inexplicable, volatile, and unpredictable behavior of groups in conflict (Volkan, 1985). The ability to signal and interpret these processes promptly may help prevent populistic political manipulations and malignant socio-political developments. However, the narcissism of minor resemblances can also enhance empathy and solidarity. It can start processes aiming at cooperation and peacebuilding, and at understanding and addressing the core causes of societal discomfort.

Moreover, on the level of individual trauma therapists assisting survivors of man-made disasters, knowledge of large-group psychological processes in general and the narcissism of minor differences and resemblances, in particular, may also be helpful. Survivors often question themselves and therapists about underlying reasons for atrocities they have been exposed to. They try to understand how their long-time neighbors have turned into enemies, how bloodshed could occur, and how and why mass violence starts and unfolds in previously peaceful communities. They try to make meaning of traumatic life events experienced to cope with feelings of powerlessness and anxiety. Other survivors may suffer from moral injury (Litz et al., 2009) related to the inner conflict arising from morally ambiguous situations occurring when someone has made decisions or witnessed actions that go against deeply held beliefs about what is right or wrong. These survivors struggle with self-forgiveness, and awareness of the concepts discussed may help them find some explanations for their experiences. Survivors may also deal with a dilemma concerning the upbringing of their children, which norms and values to transfer to the next generation, what to teach the offspring about traumatic experiences they have survived, and over norms and values of the human race in general. Knowledge about concepts of the narcissism of minor differences and resemblances may facilitate the meaning-making process in survivors and help trauma treatment.

The theoretical considerations presented in this article are based on the author’s observations and are rooted in the works of other scholars. However, the concept presented lacks scientific validation and should be further examined. Moreover, the concept of narcissism should be developed more in-depth to lead to a much-needed consensus on common terminology. The complexity of this concept and its different dimensions must be unraveled. An integrative framework for its different dimensions needs to be better understood, as the development of narcissism seems to reflect principles of equifinaity and multifinality (Cicchetti and Rogosch, 1996).

## Conclusion

The narcissism of minor resemblances is suggested to be a helpful concept for understanding large group dynamics at times of perceived uncertainty and peril. It operates side by side with the concept of the narcissism of minor differences in the process of creating enemies and allies. The narcissism of minor resemblances may help mend a large group’s narcissistic injuries, but it also serves as a defense against their self-examination. It is hypothesized that it helps repair the vulnerable aspect of wounded narcissism, while the narcissism of minor differences may serve as an antidote for the grandiose aspect.

## Data availability statement

The original contributions presented in the study are included in the article/supplementary material; further inquiries can be directed to the corresponding author.

## Author contributions

BD: Writing – original draft.
